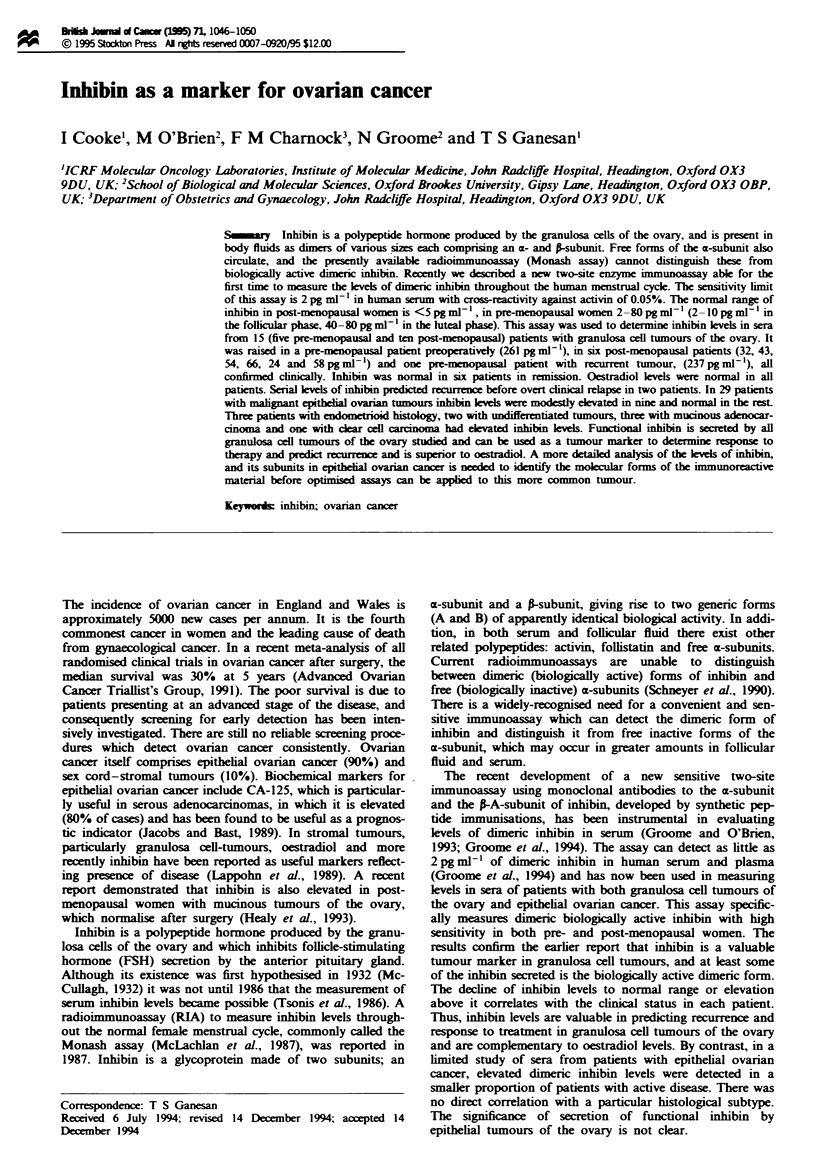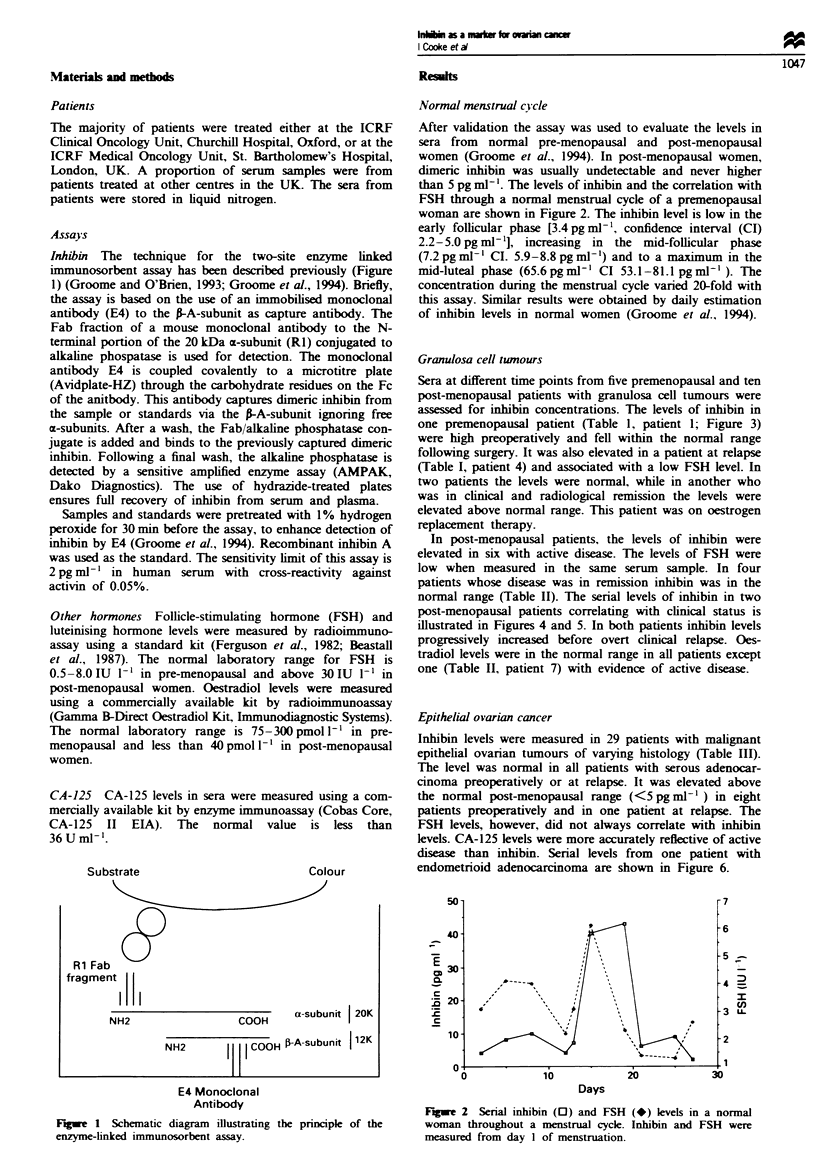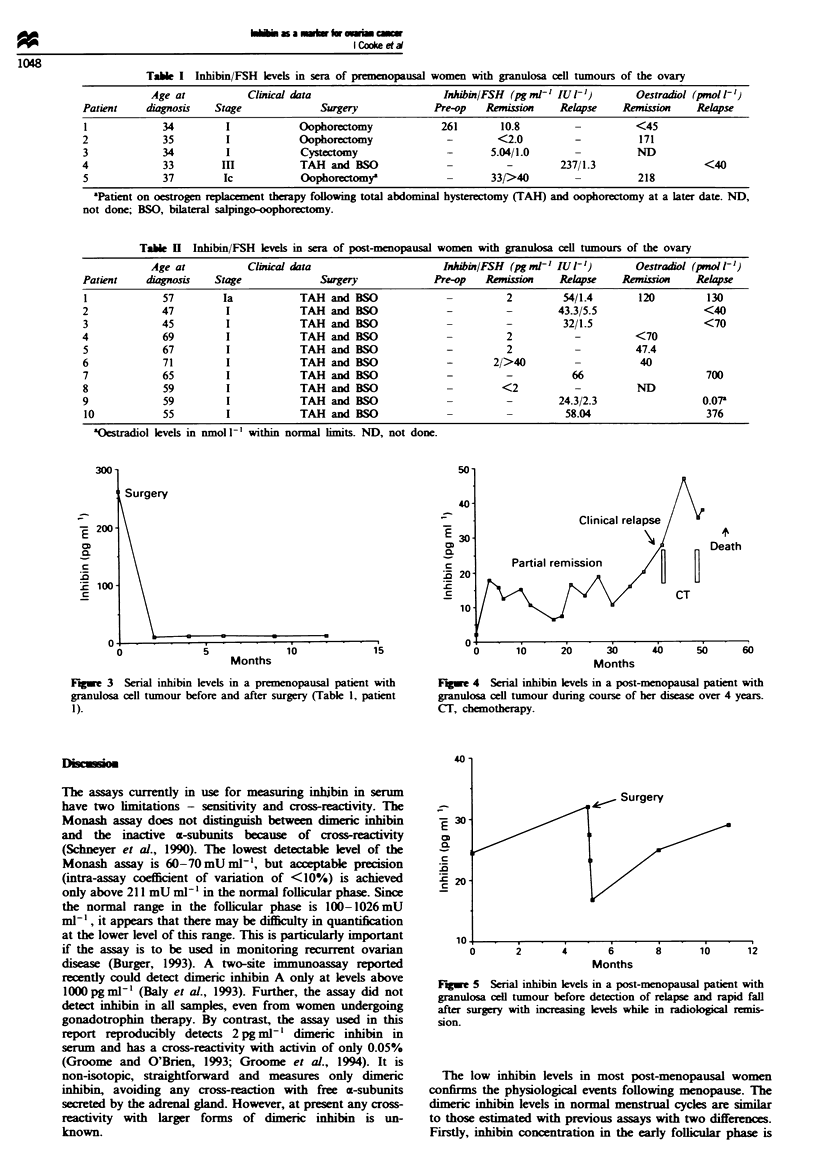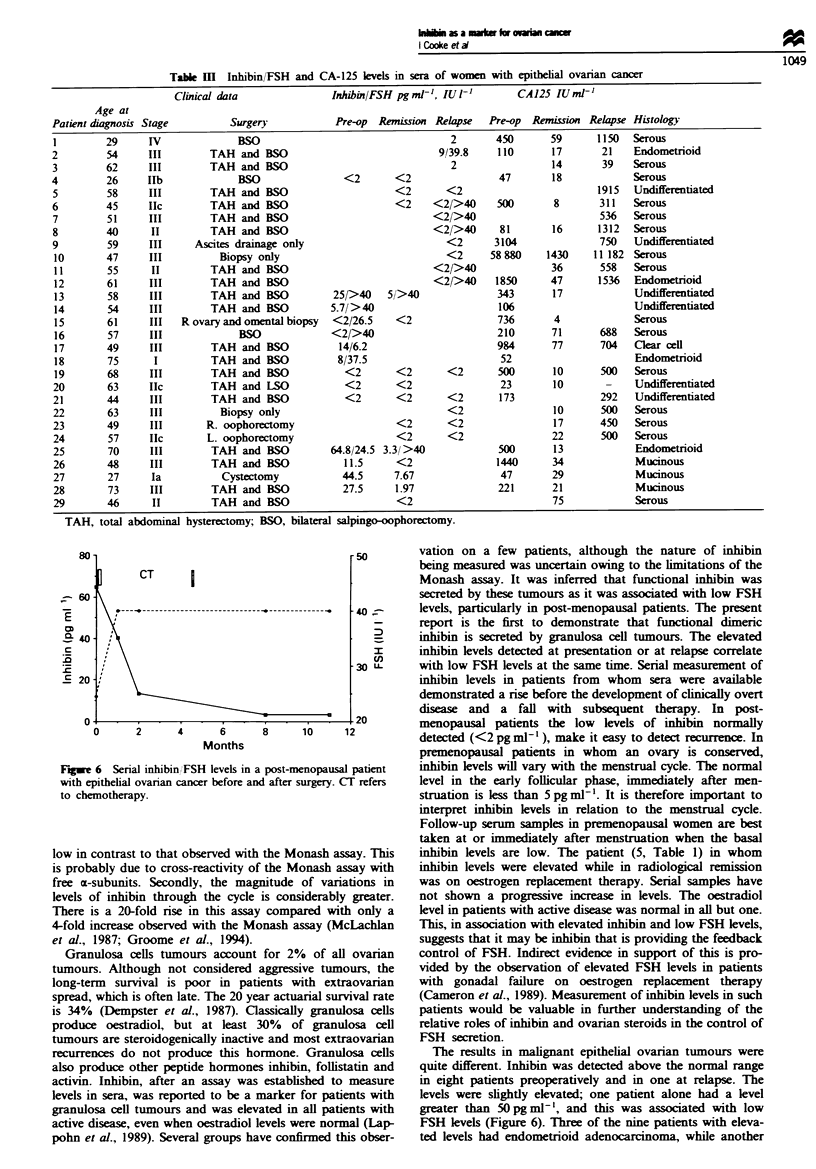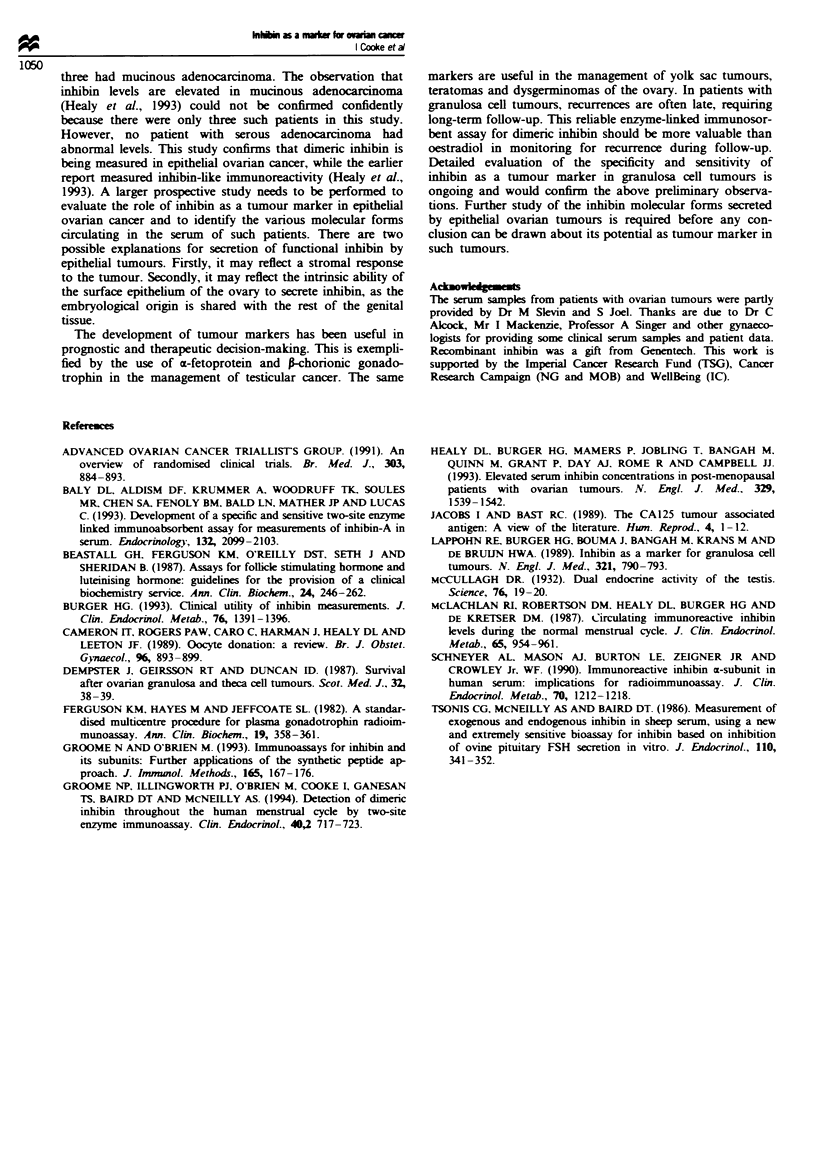# Inhibin as a marker for ovarian cancer.

**DOI:** 10.1038/bjc.1995.201

**Published:** 1995-05

**Authors:** I. Cooke, M. O'Brien, F. M. Charnock, N. Groome, T. S. Ganesan

**Affiliations:** ICRF Molecular Oncology Laboratories, Institute of Molecular Medicine, John Radcliffe Hospital, Headington, Oxford, UK.

## Abstract

Inhibin is a polypeptide hormone produced by the granulosa cells of the ovary, and is present in body fluids as dimers of various sizes each comprising an alpha- and beta-subunit. Free forms of the alpha-subunit also circulate, and the presently available radioimmunoassay (Monash assay) cannot distinguish these from biologically active dimeric inhibin. Recently we described a new two-site enzyme immunoassay able for the first time to measure the levels of dimeric inhibin throughout the human menstrual cycle. The sensitivity limit of this assay is 2 pg ml-1 in human serum with cross-reactivity against activin of 0.05%. The normal range of inhibin in post-menopausal women is < 5 pg ml-1, in pre-menopausal women 2-80 pg ml-1 (2-10 pg ml-1 in the follicular phase, 40-80 pg ml-1 in the luteal phase). This assay was used to determine inhibin levels in sera from 15 (five pre-menopausal and ten post-menopausal) patients with granulosa cell tumours of the ovary. It was raised in a pre-menopausal patient preoperatively (261 pg ml-1), in six post-menopausal patients (32, 43, 54, 66, 24 and 58 pg ml-1) and one pre-menopausal patient with recurrent tumour, (237 pg ml-1), all confirmed clinically. Inhibin was normal in six patients in remission. Oestradiol levels were normal in all patients. Serial levels of inhibin predicted recurrence before overt clinical relapse in two patients. In 29 patients with malignant epithelial ovarian tumours inhibin levels were modestly elevated in nine and normal in the rest. Three patients with endometrioid histology, two with undifferentiated tumours, three with mucinous adenocarcinoma and one with clear cell carcinoma had elevated inhibin levels. Functional inhibin is secreted by all granulosa cell tumours of the ovary studied and can be used as a tumour marker to determine response to therapy and predict recurrence and is superior to oestradiol. A more detailed analysis of the levels of inhibin, and its subunits in epithelial ovarian cancer is needed to identify the molecular forms of the immunoreactive material before optimised assays can be applied to this more common tumour.


© 1995 Stockton Press All rights reserved 0007–0920/95 $12.00

### Inhibin as a marker for ovarian cancer

I Cooke[1], M O'Brien[2], F M Charnock[3], N Groome[2] and T S Ganesan[1]

[1]*ICRF Molecular Oncology Laboratories, Institute of Molecular Medicine, John Radcliffe Hospital, Headington, Oxford OX3 9DU, UK;* [2]*School of Biological and Molecular Sciences, Oxford Brookes University, Gipsy Lane, Headington, Oxford OX3 OBP, UK;* [3]*Department of Obstetrics and Gynaecology, John Radcliffe Hospital, Headington, Oxford OX3 9DU, UK*

**Summary** Inhibin is a polypeptide hormone produced by the granulosa cells of the ovary, and is present in body fluids as dimers of various sizes each comprising an α- and β-subunit. Free forms of the α-subunit also circulate, and the presently available radioimmunoassay (Monash assay) cannot distinguish these from biologically active dimeric inhibin. Recently we described a new two-site enzyme immunoassay able for the first time to measure the levels of dimeric inhibin throughout the human menstrual cycle. The sensitivity limit of this assay is 2 pg ml$^{-1}$ in human serum with cross-reactivity against activin of 0.05%. The normal range of inhibin in post-menopausal women is <5 pg ml$^{-1}$, in pre-menopausal women 2–80 pg ml$^{-1}$ (2–10 pg ml$^{-1}$ in the follicular phase, 40–80 pg ml$^{-1}$ in the luteal phase). This assay was used to determine inhibin levels in sera from 15 (five pre-menopausal and ten post-menopausal) patients with granulosa cell tumours of the ovary. It was raised in a pre-menopausal patient preoperatively (261 pg ml$^{-1}$), in six post-menopausal patients (32, 43, 54, 66, 24 and 58 pg ml$^{-1}$) and one pre-menopausal patient with recurrent tumour, (237 pg ml$^{-1}$), all confirmed clinically. Inhibin was normal in six patients in remission. Oestradiol levels were normal in all patients. Serial levels of inhibin predicted recurrence before overt clinical relapse in two patients. In 29 patients with malignant epithelial ovarian tumours inhibin levels were modestly elevated in nine and normal in the rest. Three patients with endometrioid histology, two with undifferentiated tumours, three with mucinous adenocarcinoma and one with clear cell carcinoma had elevated inhibin levels. Functional inhibin is secreted by all granulosa cell tumours of the ovary studied and can be used as a tumour marker to determine response to therapy and predict recurrence and is superior to oestradiol. A more detailed analysis of the levels of inhibin, and its subunits in epithelial ovarian cancer is needed to identify the molecular forms of the immunoreactive material before optimised assays can be applied to this more common tumour.

**Keywords:** inhibin; ovarian cancer

The incidence of ovarian cancer in England and Wales is approximately 5000 new cases per annum. It is the fourth commonest cancer in women and the leading cause of death from gynaecological cancer. In a recent meta-analysis of all randomised clinical trials in ovarian cancer after surgery, the median survival was 30% at 5 years (Advanced Ovarian Cancer Triallist's Group, 1991). The poor survival is due to patients presenting at an advanced stage of the disease, and consequently screening for early detection has been intensively investigated. There are still no reliable screening procedures which detect ovarian cancer consistently. Ovarian cancer itself comprises epithelial ovarian cancer (90%) and sex cord–stromal tumours (10%). Biochemical markers for epithelial ovarian cancer include CA-125, which is particularly useful in serous adenocarcinomas, in which it is elevated (80% of cases) and has been found to be useful as a prognostic indicator (Jacobs and Bast, 1989). In stromal tumours, particularly granulosa cell-tumours, oestradiol and more recently inhibin have been reported as useful markers reflecting presence of disease (Lappohn *et al.*, 1989). A recent report demonstrated that inhibin is also elevated in post-menopausal women with mucinous tumours of the ovary, which normalise after surgery (Healy *et al.*, 1993).

Inhibin is a polypeptide hormone produced by the granulosa cells of the ovary and which inhibits follicle-stimulating hormone (FSH) secretion by the anterior pituitary gland. Although its existence was first hypothesised in 1932 (McCullagh, 1932) it was not until 1986 that the measurement of serum inhibin levels became possible (Tsonis *et al.*, 1986). A radioimmunoassay (RIA) to measure inhibin levels throughout the normal female menstrual cycle, commonly called the Monash assay (McLachlan *et al.*, 1987), was reported in 1987. Inhibin is a glycoprotein made of two subunits; an α-subunit and a β-subunit, giving rise to two generic forms (A and B) of apparently identical biological activity. In addition, in both serum and follicular fluid there exist other related polypeptides: activin, follistatin and free α-subunits. Current radioimmunoassays are unable to distinguish between dimeric (biologically active) forms of inhibin and free (biologically inactive) α-subunits (Schneyer *et al.*, 1990). There is a widely-recognised need for a convenient and sensitive immunoassay which can detect the dimeric form of inhibin and distinguish it from free inactive forms of the α-subunit, which may occur in greater amounts in follicular fluid and serum.

The recent development of a new sensitive two-site immunoassay using monoclonal antibodies to the α-subunit and the β-A-subunit of inhibin, developed by synthetic peptide immunisations, has been instrumental in evaluating levels of dimeric inhibin in serum (Groome and O'Brien, 1993; Groome *et al.*, 1994). The assay can detect as little as 2 pg ml$^{-1}$ of dimeric inhibin in human serum and plasma (Groome *et al.*, 1994) and has now been used in measuring levels in sera of patients with both granulosa cell tumours of the ovary and epithelial ovarian cancer. This assay specifically measures dimeric biologically active inhibin with high sensitivity in both pre- and post-menopausal women. The results confirm the earlier report that inhibin is a valuable tumour marker in granulosa cell tumours, and at least some of the inhibin secreted is the biologically active dimeric form. The decline of inhibin levels to normal range or elevation above it correlates with the clinical status in each patient. Thus, inhibin levels are valuable in predicting recurrence and response to treatment in granulosa cell tumours of the ovary and are complementary to oestradiol levels. By contrast, in a limited study of sera from patients with epithelial ovarian cancer, elevated dimeric inhibin levels were detected in a smaller proportion of patients with active disease. There was no direct correlation with a particular histological subtype. The significance of secretion of functional inhibin by epithelial tumours of the ovary is not clear.

Correspondence: T S Ganesan
Received 6 July 1994; revised 14 December 1994; accepted 14 December 1994

### Materials and methods

### Patients

The majority of patients were treated either at the ICRF Clinical Oncology Unit, Churchill Hospital, Oxford, or at the ICRF Medical Oncology Unit, St. Bartholomew's Hospital, London, UK. A proportion of serum samples were from patients treated at other centres in the UK. The sera from patients were stored in liquid nitrogen.

### Assays

*Inhibin* The technique for the two-site enzyme linked immunosorbent assay has been described previously (Figure 1) (Groome and O'Brien, 1993; Groome *et al.*, 1994). Briefly, the assay is based on the use of an immobilised monoclonal antibody (E4) to the β-A-subunit as capture antibody. The Fab fraction of a mouse monoclonal antibody to the N-terminal portion of the 20 kDa α-subunit (R1) conjugated to alkaline phospatase is used for detection. The monoclonal antibody E4 is coupled covalently to a microtitre plate (Avidplate-HZ) through the carbohydrate residues on the Fc of the anitbody. This antibody captures dimeric inhibin from the sample or standards via the β-A-subunit ignoring free α-subunits. After a wash, the Fab/alkaline phosphatase conjugate is added and binds to the previously captured dimeric inhibin. Following a final wash, the alkaline phosphatase is detected by a sensitive amplified enzyme assay (AMPAK, Dako Diagnostics). The use of hydrazide-treated plates ensures full recovery of inhibin from serum and plasma.

Samples and standards were pretreated with 1% hydrogen peroxide for 30 min before the assay, to enhance detection of inhibin by E4 (Groome *et al.*, 1994). Recombinant inhibin A was used as the standard. The sensitivity limit of this assay is 2 pg $ml^{-1}$ in human serum with cross-reactivity against activin of 0.05%.

*Other hormones* Follicle-stimulating hormone (FSH) and luteinising hormone levels were measured by radioimmunoassay using a standard kit (Ferguson *et al.*, 1982; Beastall *et al.*, 1987). The normal laboratory range for FSH is 0.5–8.0 IU $l^{-1}$ in pre-menopausal and above 30 IU $l^{-1}$ in post-menopausal women. Oestradiol levels were measured using a commercially available kit by radioimmunoassay (Gamma B-Direct Oestradiol Kit, Immunodiagnostic Systems). The normal laboratory range is 75–300 pmol $l^{-1}$ in pre-menopausal and less than 40 pmol $l^{-1}$ in post-menopausal women.

*CA-125* CA-125 levels in sera were measured using a commercially available kit by enzyme immunoassay (Cobas Core, CA-125 II EIA). The normal value is less than 36 U $ml^{-1}$.

**Figure 1** Schematic diagram illustrating the principle of the enzyme-linked immunosorbent assay.

### Results

### Normal menstrual cycle

After validation the assay was used to evaluate the levels in sera from normal pre-menopausal and post-menopausal women (Groome *et al.*, 1994). In post-menopausal women, dimeric inhibin was usually undetectable and never higher than 5 pg $ml^{-1}$. The levels of inhibin and the correlation with FSH through a normal menstrual cycle of a premenopausal woman are shown in Figure 2. The inhibin level is low in the early follicular phase [3.4 pg $ml^{-1}$, confidence interval (CI) 2.2–5.0 pg $ml^{-1}$], increasing in the mid-follicular phase (7.2 pg $ml^{-1}$ CI. 5.9–8.8 pg $ml^{-1}$) and to a maximum in the mid-luteal phase (65.6 pg $ml^{-1}$ CI 53.1–81.1 pg $ml^{-1}$ ). The concentration during the menstrual cycle varied 20-fold with this assay. Similar results were obtained by daily estimation of inhibin levels in normal women (Groome *et al.*, 1994).

### Granulosa cell tumours

Sera at different time points from five premenopausal and ten post-menopausal patients with granulosa cell tumours were assessed for inhibin concentrations. The levels of inhibin in one premenopausal patient (Table 1, patient 1; Figure 3) were high preoperatively and fell within the normal range following surgery. It was also elevated in a patient at relapse (Table I, patient 4) and associated with a low FSH level. In two patients the levels were normal, while in another who was in clinical and radiological remission the levels were elevated above normal range. This patient was on oestrogen replacement therapy.

In post-menopausal patients, the levels of inhibin were elevated in six with active disease. The levels of FSH were low when measured in the same serum sample. In four patients whose disease was in remission inhibin was in the normal range (Table II). The serial levels of inhibin in two post-menopausal patients correlating with clinical status is illustrated in Figures 4 and 5. In both patients inhibin levels progressively increased before overt clinical relapse. Oestradiol levels were in the normal range in all patients except one (Table II, patient 7) with evidence of active disease.

### Epithelial ovarian cancer

Inhibin levels were measured in 29 patients with malignant epithelial ovarian tumours of varying histology (Table III). The level was normal in all patients with serous adenocarcinoma preoperatively or at relapse. It was elevated above the normal post-menopausal range (<5 pg $ml^{-1}$ ) in eight patients preoperatively and in one patient at relapse. The FSH levels, however, did not always correlate with inhibin levels. CA-125 levels were more accurately reflective of active disease than inhibin. Serial levels from one patient with endometrioid adenocarcinoma are shown in Figure 6.

**Figure 2** Serial inhibin (□) and FSH (◆) levels in a normal woman throughout a menstrual cycle. Inhibin and FSH were measured from day 1 of menstruation.

**Table I** Inhibin/FSH levels in sera of premenopausal women with granulosa cell tumours of the ovary

| Patient | Age at diagnosis | Clinical data: Stage | Clinical data: Surgery | Inhibin/FSH ($pg\ ml^{-1}$ $IU\ l^{-1}$): Pre-op | Remission | Relapse | Oestradiol ($pmol\ l^{-1}$): Remission | Relapse |
|---|---|---|---|---|---|---|---|---|
| 1 | 34 | I | Oophorectomy | 261 | 10.8 | – | <45 | |
| 2 | 35 | I | Oophorectomy | – | <2.0 | – | 171 | |
| 3 | 34 | I | Cystectomy | – | 5.04/1.0 | – | ND | |
| 4 | 33 | III | TAH and BSO | – | – | 237/1.3 | | <40 |
| 5 | 37 | Ic | Oophorectomy[a] | – | 33/>40 | – | 218 | |

[a]Patient on oestrogen replacement therapy following total abdominal hysterectomy (TAH) and oophorectomy at a later date. ND, not done; BSO, bilateral salpingo-oophorectomy.

**Table II** Inhibin/FSH levels in sera of post-menopausal women with granulosa cell tumours of the ovary

| Patient | Age at diagnosis | Clinical data: Stage | Clinical data: Surgery | Inhibin/FSH ($pg\ ml^{-1}$ $IU\ l^{-1}$): Pre-op | Remission | Relapse | Oestradiol ($pmol\ l^{-1}$): Remission | Relapse |
|---|---|---|---|---|---|---|---|---|
| 1 | 57 | Ia | TAH and BSO | – | 2 | 54/1.4 | 120 | 130 |
| 2 | 47 | I | TAH and BSO | – | – | 43.3/5.5 | | <40 |
| 3 | 45 | I | TAH and BSO | – | – | 32/1.5 | | <70 |
| 4 | 69 | I | TAH and BSO | – | 2 | – | <70 | |
| 5 | 67 | I | TAH and BSO | – | 2 | – | 47.4 | |
| 6 | 71 | I | TAH and BSO | – | 2/>40 | – | 40 | |
| 7 | 65 | I | TAH and BSO | – | – | 66 | | 700 |
| 8 | 59 | I | TAH and BSO | – | <2 | – | ND | |
| 9 | 59 | I | TAH and BSO | – | – | 24.3/2.3 | | 0.07[a] |
| 10 | 55 | I | TAH and BSO | – | – | 58.04 | | 376 |

[a]Oestradiol levels in $nmol\ l^{-1}$ within normal limits. ND, not done.

**Figure 3** Serial inhibin levels in a premenopausal patient with granulosa cell tumour before and after surgery (Table 1, patient 1).

**Figure 4** Serial inhibin levels in a post-menopausal patient with granulosa cell tumour during course of her disease over 4 years. CT, chemotherapy.

### Discussion

The assays currently in use for measuring inhibin in serum have two limitations – sensitivity and cross-reactivity. The Monash assay does not distinguish between dimeric inhibin and the inactive α-subunits because of cross-reactivity (Schneyer *et al.*, 1990). The lowest detectable level of the Monash assay is 60–70 $mU\ ml^{-1}$, but acceptable precision (intra-assay coefficient of variation of <10%) is achieved only above 211 $mU\ ml^{-1}$ in the normal follicular phase. Since the normal range in the follicular phase is 100–1026 mU $ml^{-1}$, it appears that there may be difficulty in quantification at the lower level of this range. This is particularly important if the assay is to be used in monitoring recurrent ovarian disease (Burger, 1993). A two-site immunoassay reported recently could detect dimeric inhibin A only at levels above 1000 $pg\ ml^{-1}$ (Baly *et al.*, 1993). Further, the assay did not detect inhibin in all samples, even from women undergoing gonadotrophin therapy. By contrast, the assay used in this report reproducibly detects 2 $pg\ ml^{-1}$ dimeric inhibin in serum and has a cross-reactivity with activin of only 0.05% (Groome and O'Brien, 1993; Groome *et al.*, 1994). It is non-isotopic, straightforward and measures only dimeric inhibin, avoiding any cross-reaction with free α-subunits secreted by the adrenal gland. However, at present any cross-reactivity with larger forms of dimeric inhibin is unknown.

**Figure 5** Serial inhibin levels in a post-menopausal patient with granulosa cell tumour before detection of relapse and rapid fall after surgery with increasing levels while in radiological remission.

The low inhibin levels in most post-menopausal women confirms the physiological events following menopause. The dimeric inhibin levels in normal menstrual cycles are similar to those estimated with previous assays with two differences. Firstly, inhibin concentration in the early follicular phase is

Table III Inhibin/FSH and CA-125 levels in sera of women with epithelial ovarian cancer

| | Clinical data | | | Inhibin/FSH $pg\,ml^{-1}$, $IU\,l^{-1}$ | | | CA125 $IU\,ml^{-1}$ | | | |
|---|---|---|---|---|---|---|---|---|---|---|
| Patient | Age at diagnosis | Stage | Surgery | Pre-op | Remission | Relapse | Pre-op | Remission | Relapse | Histology |
| 1 | 29 | IV | BSO | | | 2 | 450 | 59 | 1150 | Serous |
| 2 | 54 | III | TAH and BSO | | | 9/39.8 | 110 | 17 | 21 | Endometrioid |
| 3 | 62 | III | TAH and BSO | | | 2 | | 14 | 39 | Serous |
| 4 | 26 | IIb | BSO | <2 | <2 | | 47 | 18 | | Serous |
| 5 | 58 | III | TAH and BSO | | <2 | <2 | | | 1915 | Undifferentiated |
| 6 | 45 | IIc | TAH and BSO | | <2 | <2/>40 | 500 | 8 | 311 | Serous |
| 7 | 51 | III | TAH and BSO | | | <2/>40 | | | 536 | Serous |
| 8 | 40 | II | TAH and BSO | | | <2/>40 | 81 | 16 | 1312 | Serous |
| 9 | 59 | III | Ascites drainage only | | | <2 | 3104 | | 750 | Undifferentiated |
| 10 | 47 | III | Biopsy only | | | <2 | 58 880 | 1430 | 11 182 | Serous |
| 11 | 55 | II | TAH and BSO | | | <2/>40 | | 36 | 558 | Serous |
| 12 | 61 | III | TAH and BSO | | | <2/>40 | 1850 | 47 | 1536 | Endometrioid |
| 13 | 58 | III | TAH and BSO | 25/>40 | 5/>40 | | 343 | 17 | | Undifferentiated |
| 14 | 54 | III | TAH and BSO | 5.7/>40 | | | 106 | | | Undifferentiated |
| 15 | 61 | III | R ovary and omental biopsy | <2/26.5 | <2 | | 736 | 4 | | Serous |
| 16 | 57 | III | BSO | <2/>40 | | | 210 | 71 | 688 | Serous |
| 17 | 49 | III | TAH and BSO | 14/6.2 | | | 984 | 77 | 704 | Clear cell |
| 18 | 75 | I | TAH and BSO | 8/37.5 | | | 52 | | | Endometrioid |
| 19 | 68 | III | TAH and BSO | <2 | <2 | <2 | 500 | 10 | 500 | Serous |
| 20 | 63 | IIc | TAH and LSO | <2 | <2 | | 23 | 10 | – | Undifferentiated |
| 21 | 44 | III | TAH and BSO | <2 | <2 | <2 | 173 | | 292 | Undifferentiated |
| 22 | 63 | III | Biopsy only | | | <2 | | 10 | 500 | Serous |
| 23 | 49 | III | R. oophorectomy | | <2 | <2 | | 17 | 450 | Serous |
| 24 | 57 | IIc | L. oophorectomy | | <2 | <2 | | 22 | 500 | Serous |
| 25 | 70 | III | TAH and BSO | 64.8/24.5 | 3.3/>40 | | 500 | 13 | | Endometrioid |
| 26 | 48 | III | TAH and BSO | 11.5 | <2 | | 1440 | 34 | | Mucinous |
| 27 | 27 | Ia | Cystectomy | 44.5 | 7.67 | | 47 | 29 | | Mucinous |
| 28 | 73 | III | TAH and BSO | 27.5 | 1.97 | | 221 | 21 | | Mucinous |
| 29 | 46 | II | TAH and BSO | | <2 | | | 75 | | Serous |

TAH, total abdominal hysterectomy; BSO, bilateral salpingo-oophorectomy.

**Figure 6** Serial inhibin/FSH levels in a post-menopausal patient with epithelial ovarian cancer before and after surgery. CT refers to chemotherapy.

low in contrast to that observed with the Monash assay. This is probably due to cross-reactivity of the Monash assay with free α-subunits. Secondly, the magnitude of variations in levels of inhibin through the cycle is considerably greater. There is a 20-fold rise in this assay compared with only a 4-fold increase observed with the Monash assay (McLachlan *et al.*, 1987; Groome *et al.*, 1994).

Granulosa cells tumours account for 2% of all ovarian tumours. Although not considered aggressive tumours, the long-term survival is poor in patients with extraovarian spread, which is often late. The 20 year actuarial survival rate is 34% (Dempster *et al.*, 1987). Classically granulosa cells produce oestradiol, but at least 30% of granulosa cell tumours are steroidogenically inactive and most extraovarian recurrences do not produce this hormone. Granulosa cells also produce other peptide hormones inhibin, follistatin and activin. Inhibin, after an assay was established to measure levels in sera, was reported to be a marker for patients with granulosa cell tumours and was elevated in all patients with active disease, even when oestradiol levels were normal (Lappohn *et al.*, 1989). Several groups have confirmed this observation on a few patients, although the nature of inhibin being measured was uncertain owing to the limitations of the Monash assay. It was inferred that functional inhibin was secreted by these tumours as it was associated with low FSH levels, particularly in post-menopausal patients. The present report is the first to demonstrate that functional dimeric inhibin is secreted by granulosa cell tumours. The elevated inhibin levels detected at presentation or at relapse correlate with low FSH levels at the same time. Serial measurement of inhibin levels in patients from whom sera were available demonstrated a rise before the development of clinically overt disease and a fall with subsequent therapy. In post-menopausal patients the low levels of inhibin normally detected ($<2\,pg\,ml^{-1}$), make it easy to detect recurrence. In premenopausal patients in whom an ovary is conserved, inhibin levels will vary with the menstrual cycle. The normal level in the early follicular phase, immediately after menstruation is less than $5\,pg\,ml^{-1}$. It is therefore important to interpret inhibin levels in relation to the menstrual cycle. Follow-up serum samples in premenopausal women are best taken at or immediately after menstruation when the basal inhibin levels are low. The patient (5, Table 1) in whom inhibin levels were elevated while in radiological remission was on oestrogen replacement therapy. Serial samples have not shown a progressive increase in levels. The oestradiol level in patients with active disease was normal in all but one. This, in association with elevated inhibin and low FSH levels, suggests that it may be inhibin that is providing the feedback control of FSH. Indirect evidence in support of this is provided by the observation of elevated FSH levels in patients with gonadal failure on oestrogen replacement therapy (Cameron *et al.*, 1989). Measurement of inhibin levels in such patients would be valuable in further understanding of the relative roles of inhibin and ovarian steroids in the control of FSH secretion.

The results in malignant epithelial ovarian tumours were quite different. Inhibin was detected above the normal range in eight patients preoperatively and in one at relapse. The levels were slightly elevated; one patient alone had a level greater than $50\,pg\,ml^{-1}$, and this was associated with low FSH levels (Figure 6). Three of the nine patients with elevated levels had endometrioid adenocarcinoma, while another three had mucinous adenocarcinoma. The observation that inhibin levels are elevated in mucinous adenocarcinoma (Healy *et al*., 1993) could not be confirmed confidently because there were only three such patients in this study. However, no patient with serous adenocarcinoma had abnormal levels. This study confirms that dimeric inhibin is being measured in epithelial ovarian cancer, while the earlier report measured inhibin-like immunoreactivity (Healy *et al*., 1993). A larger prospective study needs to be performed to evaluate the role of inhibin as a tumour marker in epithelial ovarian cancer and to identify the various molecular forms circulating in the serum of such patients. There are two possible explanations for secretion of functional inhibin by epithelial tumours. Firstly, it may reflect a stromal response to the tumour. Secondly, it may reflect the intrinsic ability of the surface epithelium of the ovary to secrete inhibin, as the embryological origin is shared with the rest of the genital tissue.

The development of tumour markers has been useful in prognostic and therapeutic decision-making. This is exemplified by the use of α-fetoprotein and β-chorionic gonadotrophin in the management of testicular cancer. The same markers are useful in the management of yolk sac tumours, teratomas and dysgerminomas of the ovary. In patients with granulosa cell tumours, recurrences are often late, requiring long-term follow-up. This reliable enzyme-linked immunosorbent assay for dimeric inhibin should be more valuable than oestradiol in monitoring for recurrence during follow-up. Detailed evaluation of the specificity and sensitivity of inhibin as a tumour marker in granulosa cell tumours is ongoing and would confirm the above preliminary observations. Further study of the inhibin molecular forms secreted by epithelial ovarian tumours is required before any conclusion can be drawn about its potential as tumour marker in such tumours.

**Acknowledgements**
The serum samples from patients with ovarian tumours were partly provided by Dr M Slevin and S Joel. Thanks are due to Dr C Alcock, Mr I Mackenzie, Professor A Singer and other gynaecologists for providing some clinical serum samples and patient data. Recombinant inhibin was a gift from Genentech. This work is supported by the Imperial Cancer Research Fund (TSG), Cancer Research Campaign (NG and MOB) and WellBeing (IC).

**References**

ADVANCED OVARIAN CANCER TRIALLISTS GROUP. (1991). An overview of randomised clinical trials. *Br. Med. J.*, **303**, 884–893.

BALY DL, ALDISM DF, KRUMMER A, WOODRUFF TK, SOULES MR, CHEN SA, FENOLY BM, BALD LN, MATHER JP AND LUCAS C. (1993). Development of a specific and sensitive two-site enzyme linked immunoabsorbent assay for measurements of inhibin-A in serum. *Endocrinology*, **132**, 2099–2103.

BEASTALL GH, FERGUSON KM, O'REILLY DST, SETH J AND SHERIDAN B. (1987). Assays for follicle stimulating hormone and luteinising hormone: guidelines for the provision of a clinical biochemistry service. *Ann. Clin. Biochem.*, **24**, 246–262.

BURGER HG. (1993). Clinical utility of inhibin measurements. *J. Clin. Endocrinol. Metab.*, **76**, 1391–1396.

CAMERON IT, ROGERS PAW, CARO C, HARMAN J, HEALY DL AND LEETON JF. (1989). Oocyte donation: a review. *Br. J. Obstet. Gynaecol.*, **96**, 893–899.

DEMPSTER J, GEIRSSON RT AND DUNCAN ID. (1987). Survival after ovarian granulosa and theca cell tumours. *Scot. Med. J.*, **32**, 38–39.

FERGUSON KM, HAYES M AND JEFFCOATE SL. (1982). A standardised multicentre procedure for plasma gonadotrophin radioimmunoassay. *Ann. Clin. Biochem.*, **19**, 358–361.

GROOME N AND O'BRIEN M. (1993). Immunoassays for inhibin and its subunits: Further applications of the synthetic peptide approach. *J. Immunol. Methods.*, **165**, 167–176.

GROOME NP, ILLINGWORTH PJ, O'BRIEN M, COOKE I, GANESAN TS, BAIRD DT AND MCNEILLY AS. (1994). Detection of dimeric inhibin throughout the human menstrual cycle by two-site enzyme immunoassay. *Clin. Endocrinol.*, **40,2** 717–723.

HEALY DL, BURGER HG, MAMERS P, JOBLING T, BANGAH M, QUINN M, GRANT P, DAY AJ, ROME R AND CAMPBELL JJ. (1993). Elevated serum inhibin concentrations in post-menopausal patients with ovarian tumours. *N. Engl. J. Med.*, **329**, 1539–1542.

JACOBS I AND BAST RC. (1989). The CA125 tumour associated antigen: A view of the literature. *Hum. Reprod.*, **4**, 1–12.

LAPPOHN RE, BURGER HG, BOUMA J, BANGAH M, KRANS M AND DE BRUIJN HWA. (1989). Inhibin as a marker for granulosa cell tumours. *N. Engl. J. Med.*, **321**, 790–793.

MCCULLAGH DR. (1932). Dual endocrine activity of the testis. *Science*, **76**, 19–20.

MCLACHLAN RI, ROBERTSON DM, HEALY DL, BURGER HG AND DE KRETSER DM. (1987). Circulating immunoreactive inhibin levels during the normal menstrual cycle. *J. Clin. Endocrinol. Metab.*, **65**, 954–961.

SCHNEYER AL, MASON AJ, BURTON LE, ZEIGNER JR AND CROWLEY Jr, WF. (1990). Immunoreactive inhibin α-subunit in human serum: implications for radioimmunoassay. *J. Clin. Endocrinol. Metab.*, **70**, 1212–1218.

TSONIS CG, MCNEILLY AS AND BAIRD DT. (1986). Measurement of exogenous and endogenous inhibin in sheep serum, using a new and extremely sensitive bioassay for inhibin based on inhibition of ovine pituitary FSH secretion in vitro. *J. Endocrinol.*, **110**, 341–352.